# Mortality rate and predictors among neonates admitted to a neonatal intensive care unit in Addis Ababa, Ethiopia: a retrospective follow-up study

**DOI:** 10.3389/fped.2024.1352270

**Published:** 2024-02-28

**Authors:** Genanew Kassie Getahun, Mihretu Beyene, Tsion Afework, Mitiku Desalegn, Selamawit Shita Jemberie, Tewodros Shitemaw

**Affiliations:** ^1^ Department of Public Health, Menelik II Medical and Health Science College, Addis Ababa, Ethiopia; ^2^ Department of Public Health, Yanet Health and Business College, Addis Ababa, Ethiopia; ^3^ Department of Anesthesia, Wachemo University, Wachemo, Ethiopia; ^4^ Department of Midwifery, College of Health Sciences, Debre Markos University, Debre Markos, Ethiopia

**Keywords:** neonatal mortality rate, predictors, retrospective follow-up, neonatal intensive care unit, Ethiopia

## Abstract

**Introduction:**

Addressing neonatal mortality is an important priority for improving the health and well-being of newborns. Almost two-thirds of infant deaths occur in the first month of life; among these, more than two-thirds die in their first week. Therefore, the aim of this study was to assess the mortality rate and predictors of mortality among neonates admitted to the neonatal intensive care unit (NICU) at Tikur Anbessa Specialized Hospital in Addis Ababa, Ethiopia, in 2023.

**Methods:**

An institutional-based retrospective follow-up study was conducted using 459 neonates who were admitted to the NICU at Tikur Anbessa Specialized Hospital from January 2020 to December 2022. The data were extracted from randomly selected charts using a pretested data extraction checklist. The Nelson Alan curve with log-rank test was used to compare the presence of differences in the mortality rate of different groups over different categorical variables. The cox proportional hazards analysis model was used to identify predictors of neonatal death. The presence and absence of statistical significance was considered at a *p*-value of less than 0.05 and the strength of association was measured using AHR.

**Results:**

The neonatal mortality rate was 3.1 (95% CI: 1.3–4.9) per 1,000 neonate-days. Low birth weight (AHR = 1.44: 95% CI: 1.06–3.13), exclusive breast-feeding (AHR = 0.74: 95% CI: 0.35–0.95), and time of exclusive breast-feeding (AHR = 0.92: 95% CI: 0.49–0.99) were the identified predictors of newborn mortality.

**Conclusion:**

The neonatal mortality rate was high. Low birth weight of the neonate, exclusive breast-feeding initiation, and time of exclusive breast-feeding were independent predictors of neonatal death. Therefore, empowering mothers to exclusively breastfeed their children, which is a cost-effective, safe, and realistic option, can significantly minimize infant mortality.

## 1 Introduction

The neonatal phase (the first 28 days of life) is the most critical period for a child's survival (1). Even though being a newborn is not an illness, many children die soon after delivery, many of them in the first four weeks of life (neonatal deaths), and the majority of those in the first week of life (early neonatal deaths) (2). Every day, an estimated 6,500 newborn fatalities occur worldwide, and one-third of all newborn deaths occur within the first day of life, with the remaining three-quarters happening within the first week (3).

According to global estimates, 2.4 million neonatal deaths (nearly 6,700 neonatal deaths per day) occurred in 2019, accounting for 44% of all fatalities among children under the age of five (4, 5), of which 98% occurred in developing countries. Per 1,000 live births, the rate of death in developing and developed countries was 28.2 and 3.5, respectively (6). The rate of newborn mortality is also unequally spread between different regions and countries (7); for instance, a child born in Sub-Saharan Africa is ten times more likely to die than a child who is born in a high-income country (8).

This neonatal period is a highly vulnerable time for the neonate, who is completing many of the physiological adjustments required for extra-uterine existence (9–11). Despite the fact that neonatal mortality is declining at a slower rate than post-neonatal and under-five mortality, success in lowering the death of newborns under the age of one month has been insignificant (12).

Neonatal death in Ethiopia decreased from 39 to 29 between 2005 and 2016, but has increased to 33 in 2019 (13), which contributed to 42% of under-five mortality (14). However, a 2020 national report estimated that neonatal mortality in Ethiopia is reduced to 27 deaths per 1,000 live births (15), suggesting that there is still much work to be done to accomplish the sustainable development goal of 12/1,000 live births (16). Moreover, according to WHO estimates, the major causes of newborn deaths in Ethiopia includes pregnancy and delivery-associated events (30%), premature birth problems (26%), sepsis (18%), congenital abnormalities (11%), and pneumonia (8%) (17).

The incidence of neonatal mortality in Ethiopia was reported to be 25.8 deaths per 1,000 neonate days in North East Ethiopia (18), 1.3 per 1,000 live births in Southern Ethiopia (19), and 62.5 per 1,000 live births in Northern Ethiopia (20). Several predictors of newborn mortality have been identified in Ethiopia, including being born from a diabetes mellitus (DM) mother, a previous history of neonatal death, respiratory distress syndrome (RDS), ANC follow-up, neonatal sepsis, marital status, birth weight at admission, gestational age less than 28 weeks, complications at birth, delayed initiation of breast-feeding, home delivery, birth interval, mode of delivery, hypoglycemia, age of neonate at admission, being born from pre-eclampsia/eclamptic mothers, and being extremely low birth weight (21–23).

Despite several initiatives and implementations targeted at lowering neonatal mortality in Ethiopia, it remains high and has not been reduced as expected. Furthermore, knowing the context-specific determinants of neonatal mortality and morbidity is critical for reducing newborn death. Therefore, the aim of this study was to assess the mortality rate and predictors of neonatal mortality among neonates admitted to the NICU of Tikur Anbessa Specialized Hospital, Addis Ababa, Ethiopia, in 2023.

## 2 Methods

### 2.1 Study area and period

The study was carried out at the Tikur Anbessa specialized hospital in Addis Ababa, Ethiopia. Addis Ababa is the capital city of Ethiopia and the largest city, with an estimated population of 5,005,524. Tikur Anbessa Specialized Hospital (TASH) is the largest public teaching hospital in Addis Ababa, Ethiopia. The Tikur Anbessa Specialized Hospital NICU ward can accommodate a maximum of 60 patients with an average of 20–40 patients' daily admission. The data was collected from July 1st to July 30th, 2023.

### 2.2 Study design and population

A retrospective follow-up study was conducted among neonates who were admitted to the Neonatal Intensive Care Unit (NICU) in Tikur Anbessa Specialized Hospital from January 2020 to December 2022.

### 2.3 Eligibility criteria

All non-surgical neonates who were admitted to the Neonatal Intensive Care Unit (NICU) in Tikur Anbessa Specialized Hospital from January 2020 to December 2022 were included. However, a neonate who was admitted to the NICU but who had incomplete charts and surgical causes of NICU admitted cases were excluded from the study.

### 2.4 Sample size determination and sampling procedure

The sample size was determined by using the double population proportion formula in the Epi Info Stat calc program by considering the following assumptions: 95% CI, power 80%, ratio of unexposed to exposed: 1:2, and parameters: outcome in exposed (gestational age < 37 weeks): 22.2% (24), and 10% incomplete data, giving a total of 466. Outcome in unexposed (gestational age > 37 weeks) = 5.42% and risk ratio (RR) = 4.09.

There were 824, 647, and 541 cases during 2020, 2021 and 2022 respectively. Using a population proportion allocation we selected a total of 191, 150 and 125 cases from each years respectively using a simple random sampling technique.

### 2.5 Study variables

Neonatal death (event or censored) was considered an outcome variable, and the independent variables were categorized as socio-demographic factors, maternal factors, neonatal factors, and obstetrical factors.

### 2.6 Data collection procedure

Data were extracted using a pretested and structured checklist developed after reviewing previous published articles (25, 26) with a relevant modification. The extraction checklist had five components including socio-demographic characteristics, maternal obstetric factors, neonatal factors, follow-up information, and the overall neonatal status. It was pretested with twenty three neonates admitted and treated at St. Paul's Hospital Mellinium Medical College neonatal intensive care unit. The data were extracted by three professional nurses and supervised by the principal investigators. Data extractors were trained for one day before the actual data collection period. Every day, after data collection, each checklist was reviewed and checked for completeness and consistency.

### 2.7 Data processing and analysis

Data was cleaned, edited, coded, entered into Epi-Info software, and exported to SPSS version 26. The Cox-proportional hazard model was used to identify predictors of neonatal mortality rate in the NICU. The strength of association was measured using adjusted hazard ratio, and reported along with a 95% CI and a *p*-value.

Mortality rate was compared using Nelson-Alan curves together with log rank test over differences categorical variables. The determinants of neonatal mortality were first screened using a bi-variable Cox regression analysis model. Then variables with a *p*-value less than 0.25 were chosen as candidate variables for the multi-variable Cox regression model.

## 3 Results

### 3.1 Socio-demographic characteristics

A total of 459 complete records were included in the study, with a response rate of 98.5%. The majority of 431 (94.5%) neonates were aged less than 7 days. Nearly half (50.9%) of the neonates were female. Regarding the age of the mothers, more than three-fourths (397, or 87.1%) of them were aged between 18 and 34 years. The vast majority 432 (94.7%) of mothers had a history of previous neonatal deaths. Moreover, 439 (96.3%) and 405 (88.8%) of mothers had ANC follow-up 1st and 4th ANC visits, respectively (Table 1).

**Table 1:** Socio-demographic characteristics of the study participants.

Variables	Categories	Censored	Event
Age of neonate in day	≤7	417 (91.4%)	14 (3.1%)
	>7	25 (5.5%)	0 (0%)
Sex of the neonate	Male	214 (46.9%)	10 (2.2%)
	Female	228 (50.0%)	4 (0.9%)
Age of the mother	18–34	385 (84.4%)	12 (2.7%)
	≥35	57 (12.5%)	2 (0.4%)
Marital status of the mother	Single	9 (2%)	2 (0.4%)
	Married	426 (93.4%)	12 (2.6%)
	Widowed	7 (1.5%)	0 (0%)
Previous history of neonatal death	Yes	22 (4.8%)	2 (0.4%)
	No	420 (92.1%)	12 (2.6%)
ANC follow-up	Yes	427 (93.6%)	12 (2.6%)
	No	15 (3.3%)	2 (0.4%)
Number of visits	Frist	0 (0%)	2 (0.4%)
	Second Visit	7 (1.5%)	0 (0%)
	Third visit	42 (9.2%)	0 (0%)
	Fourth visit	393 (86.2%)	12 (2.6%)
Previous history of pregnancy	Yes	313 (68.6%)	12 (2.6%)
	No	129 (28.3%)	2 (0.4%)
The number of pregnancies before this birth	0	116 (25.4%)	2 (0.4%)
	1	120 (26.3%)	6 (1.3%)
	2	101 (22.1%)	4 (0.9%)
	3	56 (12.3%)	2 (0.4%)
	4	41 (9.0%)	0 (0%)
	5	6 (1.3%)	0 (0%)
	11	2 (0.4%)	0 (0%)

### 3.2 Obstetrical and neonatal factors

Three hundred eighty-seven (84.9%) of the neonates had five APGAR scores, and only one fifth of them (20.3%) had an APGAR score of 7–10. With regard to the weight at birth, 178 (39%) of neonates weighed <2,500 g. The majority of 289 (63.4%) of the neonates had initiated exclusive breast feeding within the first hour after birth. Concerning the mode of neonatal delivery, 282 (61.8%) of them were delivered via spontaneous vaginal delivery, and 170 (37.3%) of them were delivered by cesarean section, whereas the left was delivered by assisted instrumental delivery (Table 2).

**Table 2:** Obstetric and neonatal factors.

Variables	Categories	Censored	Event
5th APGAR score	Yes	373 (81.8%)	14 (3.1%)
	No	69 (15.1%)	0 (0%)
Score of the APGAR	0–3	58 (13.4%)	0 (0%)
	4–6	15 (3.5%)	2 (0.4%)
	7–10	346 (79.9%)	12 (2.8%)
Weight at birth (in grams)	<2,500	174 (38.2%)	4 (0.9%)
	2,500–4,000	250 (54.8%)	10 (2.2%)
	>4,000	18 (3.9%)	0 (0%)
Time of EBF	Within an hour	281 (61.6%)	8 (1.8%)
	After 1 h	161 (35.3%)	6 (1.3%)
Congenital anomaly	Yes	69 (15.1%)	6 (1.3%)
	No	373 (81.8%)	8 (1.8%)
Birth asphyxia	Yes	95 (20.8%)	2 (0.4%)
	No	347 (76.1%)	12 (2.6%)
Mode of delivery	SVD	276 (60.5%)	6 (1.3%)
	CS	164 (36.0%)	6 (1.3%
	Instrumental	2 (0.4%)	2 (0.4%))
Type of pregnancy	Single tone	409 (89.7%)	12 (2.6%)
	Twin	31 (6.8%)	2 (0.4%)
	Triple	2 (0.4%)	0 (0%)

**Figure 1:**
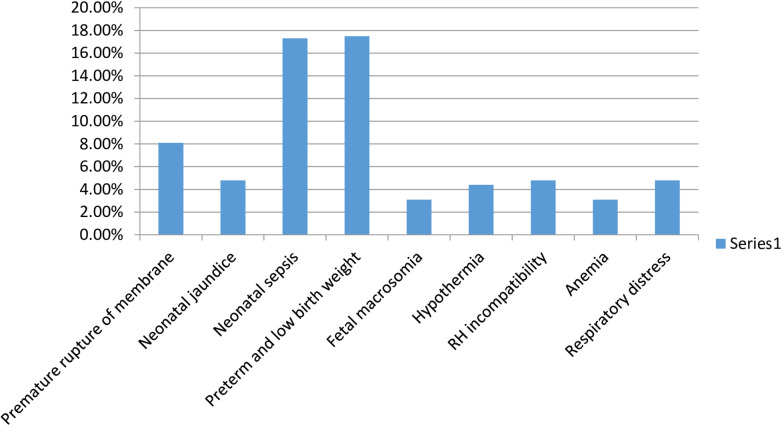
Diagnosis during admission.

Moreover, concerning the diagnosis during admission, the most common causes were neonatal sepsis 83 (18.1%), premature rupture of membranes 39 (8.5%), respiratory distress 45 (9.8%), preterm and low birth weight 80 (17.4%), and RH incompatibility 22 (4.8%) (Figure 1).

### 3.3 Neonatal mortality rate

According to the finding of this study, neonatal mortality rate was 3.1 (95% CI: 1.3–4.9), per 1,000 neonates-days.

### 3.4 Predictors associated with neonatal mortality rate

Based on the result of a bi-variable cox proportional hazard regression analysis, predictors like age of neonate in day, sex of the neonate, previous history of neonatal death, ANC follow-up, previous history of pregnancy, APGAR score, birth weight, exclusive breast-feeding initiation, and time of exclusive breast feeding were the predictors of neonatal mortality with a *p*-value less than 0.25.

Furthermore, the multi-variable cox proportional hazard regression analysis revealed that only low birthweight, exclusive breast-feeding, and exclusive breast feeding initiation time were the predictors of neonatal mortality rate. Subsequently, low birth weight newborns had a 44% higher risk of developing neonatal mortality compared with their counter peers (AHR = 1.44: 95% CI: 1.06–3.13). On the other hand exclusive breast feeding were protective against neonatal mortality. Those neonates who were exclusive breast feeding had a 26% lower risk of developing neonatal mortality compared with its counter parts (AHR = 0.74: 95% CI: 0.35–0.95). Moreover, those mothers who initiated exclusive breast feeding within the first one hour had 18% lower risk of neonatal mortality compared with its counter parts (AHR = 0.92: 95% CI: 0.49–0.99) (Table 3).

**Table 3:** The predictors of neonatal mortality rate.

Variables		Status of the neonate		Cox regression	
		Event	Censored	CHR [95% CI]	AHR [95% CI]
Age of neonate in day	≤7	13	417	.93 (.12–7.50)	–
	>7	1	25	1	1
Sex of the neonate	Male	10	214	1.69 (.46–6.24)	0.62 (0.07–5.62)
	Female	4	228	1	1
Previous history of neonatal death	Yes	2	22	.542 (.19–1.53)	0.45 (0.09–0.92)
	No	12	420	1	1
ANC follow-up	Yes	12	427	.72 (.33–1.58)	0.83 (0.03–21.52)
	No	2	15	1	1
Previous history of pregnancy	Yes	12	313	1.41 (.65–3.03)	2.83 (0.33–24.47)
	No	2	129	1	1
APGAR score	4–6	2	15	1	1
	7–10	12	346	.52 (.11–2.51)	–
Birth weight at birth	<2,500	4	174	1.78 (.91–3.50)	1.44 (1.06–3.13)*
	2,500–4,000	10	250	1	1
Exclusive breast-feeding initiation	Yes	6	301	.62 (.34–1.14)	0.74 (0.35–0.95)*
	No	8	301	1	1
Time of exclusive breast feeding initiation among exclusive breast feeding mothers	Within an hour	6	142	.7 (.44–1.38)	0.92 (0.49–0.99)*
	After 1 h	8	151	1	1

* Indicates a *p*-value less than 0.05.

## 4 Discussion

The results of this study showed that the neonatal mortality rate was 3.1 (95% CI: 1.3–4.9) per 1,000 neonate-days. This study finding was consistent with a study conducted in Kersa district, Ethiopia, which showed that the overall neonatal mortality rate was 28.37 per 1,000 live births (27). Furthermore, this study finding was in line with a study conducted in Uganda, which elucidated that the incidence of neonatal mortality in Uganda was 30 deaths per 1,000 live births (3 per 100 person days) (28). Additionally, this study finding was consistent with the study conducted at Fallujah General Hospital, which showed that the neonatal mortality rate was 50.3/1,000 of total births, while the neonatal mortality was 41.5/1,000 of total live births in the same area (29).

However, this study finding was lower than the study conducted in the rural northern part of Ethiopia, and Tigray Ethiopia which revealed that neonatal mortality rate was 18.6 per 1,000 neonate days and 62.5 respectively (30). This inconsistency might be due to the differences in study period, study setting and healthcare service improvements over time. The fact that this study was carried out in Ethiopia's capital city might have contributed to the reduced rate of neonatal mortality because of the existence of comprehensive and specialized hospitals like Black lion.

The findings of this study also showed that those neonates with a birth weight of <2,500 grams were 1.44 times more likely to have a high risk of dying than their counter peers (AHR1.44; 95% CI: 1.06–3.13). This study finding was consistent with a study conducted in India and Brazil which showed that neonates born with a normal birth weight (2,500–3,500 g) had a 55% lower hazard of neonatal death compared to neonates born with birth weights of less than 2,500 g (low birth weight) (31, 32). The possible justification behind this might be due to the fact that low birth weight is linked to a variety of negative health outcomes, including prenatal and neonatal mortality and morbidity, impaired growth and cognitive development, and even a non-communicable diseases risk later in their life.

Neonatal mortality was also associated with exclusive breast feeding initiation and the time of exclusive breast feeding. Those newborns who initiated exclusive breast feeding on time had a 36% lower risk of neonatal death compared with their counterparts. This finding was supported by similar studies from the Northwest and Bahir Dar, Ethiopia (33, 34). This might be due to the fact that breast milk is the ideal nutrient for a newborn and is easily digestible, absorbable, and metabolized, promoting bonding, improved behavioral and neurodevelopment, protecting against various infectious diseases, and promoting long-term health, which ultimately reduces neonatal mortality if practiced optimally.

### 4.1 Strengths and limitations of the study

This study's merits include the use of a relatively larger sample sizes and statistical analyses relevant to the study design. On the other hand, the study was conducted in a hospital; as a result, newborns that were born and died at home may have been overlooked. Therefore, this study does not reflect population-based neonatal mortality.

## 5 Conclusion

The neonatal mortality rate was high. Weight of the neonate, exclusive breast feeding initiation, and time of exclusive breast feeding were independent predictors of neonatal death. Therefore, empowering mothers to breastfeed their children exclusively, which is a cost-effective, safe, and feasible method, can significantly reduce infant mortality.

## Data Availability

The original contributions presented in the study are included in the article/Supplementary Material, further inquiries can be directed to the corresponding author.
